# Complications and Healthcare Consumption of Pregnant Women with a Migrant Background: Could There be an Association with Psychological Distress?

**DOI:** 10.1007/s10995-022-03451-0

**Published:** 2022-06-02

**Authors:** Hanna M. Heller, Annemijn V. R. de Vries, Adriaan W. Hoogendoorn, Fedde Scheele, Willem J. Kop, Christianne J. M. de Groot, Adriaan Honig, Birit F. P. Broekman

**Affiliations:** 1 Department of Psychiatry, Amsterdam UMC Location Vrije Universiteit Amsterdam, Boelelaan 1117, Amsterdam, The Netherlands; 2 Amsterdam Public Health, Mental Health Program, Van der Boechorststraat 7, 1081 BT Amsterdam, The Netherlands; 3 Department of Psychology and Psychiatry, Spaarne Gasthuis, POB 417, 2000 AK Haarlem, The Netherlands; 4 GGZ inGeest Mental Health Care, 1081 HJ Amsterdam, The Netherlands; 5 Department of Obstetrics and Gynaecology, OLVG Hospital, Jan Tooropstraat 164, 1061 AE Amsterdam, The Netherlands; 6 Department of Obstetrics and Gynaecology, Amsterdam UMC Location Vrije Universiteit Amsterdam, Boelelaan 1117, 1081 HV Amsterdam, The Netherlands; 7 Department of Medical and Clinical Psychology, Tilburg University, De Warandelaan 2, 5037 AB Tilburg, The Netherlands; 8 Amsterdam Reproduction & Development, Boelelaan 1117, 1081 HV Amsterdam, The Netherlands; 9 Department of Psychiatry, OLVG Hospital, Jan Tooropstraat 164, 1061 AE Amsterdam, The Netherlands

**Keywords:** Pregnancy, Psychological distress, Migrant, Complications, Healthcare consumption

## Abstract

**Objective:**

Previous studies reported less prenatal healthcare consumption and more perinatal complications in women with a migrant background. Hence, we investigated in a country with free healthcare access whether women with a migrant background differed with respect to pregnancy complications, healthcare consumption and in terms of associations with psychological distress in comparison to native Dutch.

**Methods:**

We included 324 native Dutch and 303 women with a migrant background, who visited two hospitals in Amsterdam for antenatal care between 2014 and 2015. Participants completed the Edinburgh Postnatal Depression Scale, the Hospital Depression and Anxiety Scale, and sociodemographic questions. Complications and healthcare consumption during pregnancy were extracted from medical records. Regression analyses were used with adjustment for covariates.

**Results:**

Except for gestational diabetes [adjusted OR = 3.09; 95% CI = (1.51, 6.32)], no differences were found between groups in perinatal complications [OR = 1.15; 95% CI = (0.80, 1.64)], nor in healthcare consumption [OR = 0.87; 95% CI = (0.63, 1.19)].

Women with a migrant background reported more depressive symptoms [Cohen’s d = 0.25; 95% CI = (0.10, 0.41)], even after adjustment for socio-economic factors. Psychological distress was associated with more hospital admissions during pregnancy. When experiencing depressive symptoms, women with a migrant background had an increased risk to be admitted [OR = 1.11; 95% CI = (1.01, 1.21)].

**Conclusions for Practice:**

This cohort study found no differences in pregnancy-related complications, except for diabetes, nor different healthcare consumption, in women with a migrant background versus native Dutch, in a country with free health care access. However, women with a migrant background experienced more depressive symptoms, and when depressed their risk for hospital admission increased. Additional research is warranted to improve healthcare for this population.

## Significance Statement

Previous studies described that women with a migrant background experience more perinatal complications, less healthcare consumption and more psychological distress.

In this study in a country with free healthcare access, women with a migrant background did not differ from native Dutch in healthcare consumption or perinatal complications, except for gestational diabetes. However depression was more common in women with a migrant background, and increased their risk for hospital admissions during pregnancy.

## Introduction

Women with a migrant background living in high income countries often experience adverse pregnancy outcomes in comparison to native women (Almeida et al., 2013; Jardine et al., 2021; Ravelli et al., 2011), although some studies did not find this association (David et al., 2017).

One reason could be that a large proportion of women with a migrant background consume less prenatal healthcare (Dowswell et al., 2015; Heaman et al., 2013), which may be related to healthcare organisation (Posthumus et al., 2017).

Second, epidemiological studies show that a large part of women with a migrant background have a higher prevalence of psychological distress (depressive and/or anxiety symptoms) than their native counterparts, not only in pregnancy (de Wit et al., 2008; Missine & Bracke, 2012) which is frequently associated with poor pregnancy outcomes (Almeida et al., 2018; Broekman et al., 2014; Grigoriadis et al., 2018). This variation in psychological distress can be explained by multiple factors including differences in socioeconomic background (de Wit et al., 2008; Missine & Bracke, 2012) social/partner support (Anderson et al., 2017), discrimination (Malmusi et al., 2017) and acculturation (Haverkamp et al., 2015). However, if psychological distress moderates the relation between migrant background and perinatal complications remains inconclusive.

To the best of our knowledge only two studies from different countries reported on the association of migrant background with psychological distress and pregnancy outcomes (Grobman et al., 2018; Hermon et al., 2019). However, in the USA the healthcare system is different, and in both countries the migrant backgrounds are different from the Netherlands. In the Netherlands medical insurance is available and compulsory for all, which offers every woman and child the chance to get high quality care, irrespective of income or social class.

The aims of this study are: (1) To compare the frequency of perinatal complications and the quantity of healthcare consumption of women with a migrant background to native Dutch women (2) To assess the prevalence of psychological distress (symptoms of depression and anxiety) in pregnant women with a migrant background compared to the native Dutch population and (3) To investigate the effect of migration background on the relation between psychological distress and perinatal complications and healthcare consumption.

## Material and Methods

### Study Design and Participants

This prospective cohort study was conducted in a university hospital in Amsterdam (Amsterdam UMC) and a general teaching hospital in Amsterdam (OLVG) to include a broad selection of women. All patients from both backgrounds were provided care in the hospital, either by a midwife or a gynaecologist, because of pregnancy complications in either the current or the previous pregnancy and occasionally on special request of the woman. Between November 2014 and November 2015 all women with a gestational age of 5 until 41 weeks visiting one of both hospitals for obstetric care were invited to participate. Women were enrolled if they were pregnant, at least 18 years old, and had sufficient knowledge of the Dutch or English language to complete the questionnaires. Before enrolment all participants provided written informed consent. The study protocol, information brochure, and informed consent form were approved by the Medical Ethics Committee of the VU University Medical Center (registration number 2014.195) and of the OLVG hospital (registration number ACWO 15u.082/DMP/15-019).

### Measures

All participants were invited to fill in questionnaires containing data on demographics, general health, previous pregnancies, and depressive and anxiety symptoms.

Questions on demographics included: age, country of birth, country of birth of the parents of the respondent, level of education and employment status. Level of education was classified according to the Dutch Standard Classification of Education: 2006—Edition 2016/’17 in three levels (level 1: generally or primary education, job specific training and lower levels of high school education, level 2: high school and senior job specific training, level 3: higher vocational and university education (CBS, 2016). Data on health and previous pregnancies included length of gestation at the time of completing the questionnaire, history of pregnancies, smoking behaviour during pregnancy, history of previous professional help for psychological problems and use of (psychopharmacological) medication.

Symptoms of depression were measured with the self-reported Edinburgh Postnatal Depression Scale (EPDS), which has a good internal consistency (Cronbach’s α = 0.82–0.84). An antenatal cut-off of 10 could be used to indicate possible depression, in concordance with other studies (Bergink et al., 2011).

Symptoms of anxiety were measured with the anxiety subscale of the Hospital Depression and Anxiety Scale (HADS-A), which is a reliable scale with good internal consistency (Cronbach’s α 0.81–0.84). An optimal cut-off of 8 could be used (Olsson et al., 2005). Normal distributions of the anxiety and depression symptoms were not checked, since sample sizes were large enough to rely on the central limit theorem that makes that the sample means approximately follow normal distributions.

In order to determine migrant status the definition of Statistics Netherlands (CBS) was used (CBS, 2012/2016). Here an individual is considered to have a migrant background when he or she (first generation) or at least one of his or her parents (second generation) was born in a country other than the Netherlands (CBS, 2012/2016; Stronks et al., 2009). We included education and employment as covariates.

After delivery, follow-up data were collected from the electronic medical records on complications during pregnancy and delivery, including preeclampsia (systolic bloodpressure ≥ 140 mmHg and/or diastolic bloodpressure ≥ 90 mmHg, measured two times after 20 weeks of pregnancy combined with proteinuria ≥ 300 mg/24 h, gestational diabetes (75 g post-load blood glucose level ≥ 7 mmol/l), preterm birth (gestational age < 37 weeks), small for gestational age (S.G.A.; weight ≤ p10) and all assisted delivery (caesarean section and operative vaginal delivery). Additionally, a composite score of the occurrence of all perinatal complications was calculated to raise statistical power for complications with a low incidence. Definitions were according to the guidelines of the Dutch Association of Gynaecologists and Obstetricians and the Dutch National Birth Register (Lips et al., 2018; Mol, 2011, 2012; Terwisscha van Scheltinga, 2017; Visser et al., 2009).

Healthcare consumption was measured by collecting data from the electronic medical records on hospitalisation during pregnancy and extended hospitalisation after delivery, because they represented the most clear and reliable notated variables of healthcare consumption. From this information a composite measure of health care use was created.

### Sample Size

Eight hundred and three (803) patients were invited, 115 refused participation (14.3%) and 61 (7.6%) were excluded due to insufficient mastery of Dutch or English language (n = 59), not being pregnant anymore (n = 1), and missing data on migrant background (n = 1). As the majority of non-participating women did not provide informed consent to obtain data from their medical records we could not compare them with the participating women with regard to perinatal complications and healthcare consumption. Hence, 627 women (78.1% of the 803 invited women) were included for analyses: 244 (38.9%) from the teaching hospital and 383 (61.1%) from the university hospital in Amsterdam. No differences were found in number of participants with migrant background (45.9% versus 49.9%, p = 0.332), between the two hospitals.

### Statistical Analysis

Pregnancy-related outcome variables included all perinatal complications and the composite score of these complications and the composite score of health care use, as described in the method section. Chi^2^ tests were used to test differences in categorical variables between native Dutch and women with migrant background.

T-tests and linear regression models were used to test differences in mean scores in “psychological distress” (which concerned symptoms of depression and anxiety, measured with the EPDS and HADS-A) between women with a migrant background and native Dutch women, with adjustment for clinical and sociodemographic variables.

Logistic regression models for complications and healthcare consumption were used to estimate differences between native women and women with a migrant background in terms of odds ratios, both unadjusted and adjusted for common confounders like socioeconomic status and Body Mass Index (BMI). Analyses were carried out on available data indicating the number of included cases, not using special techniques to deal with missing data.

SPSS version 24 was used for data analysis. A two-sided p value < 0.05 was considered statistically significant.

## Results

### Patient Sample

In total, 324 (51.7%) women were native Dutch and 303 (48.3%) from migrant background (see Table 1), which reflects the composition of people with a migrant background in Amsterdam (Hylkema et al., 2014). 180 women (28.7%) were first generation and 123 second generation migrants (19.6%). Native pregnant women were more often of higher maternal age, more often employed, had higher educations, lower BMI’s, sought more professional help for psychological problems and used more often psychiatric medication than pregnant women with a migrant background. No differences were observed in terms of living with a partner, parity, smoking in pregnancy or the use of previous mental health care. We found no differences in affective symptoms, complications or hospitalisation in pregnancy between the hospitals. Extended hospitalisation after delivery differed between the hospitals [OR = 1.45; 95% CI = (1.014, 2.083)], but was not moderated by migrant background.

**Table 1:** Baseline characteristics of the total sample (n = 627), of the native Dutch women (n = 324, 51.7%) and women with migrant background (n = 303, 48.3%) subsamples and p values on test of independence

	Total sample n (%)	Dutch n (%)	Migrant background n (%)	p value Chi^2^- or t-test
Maternal age, years (SD) (n = 627)	32.7 (4.9)	33.4 (4.7)	31.9 (4.9)	< 0.001
Living with partner (n = 469)	446 (95.1%)	238 (96.4%)	208 (93.7%)	0.182
Employed (n = 626)	463 (74.0%)	281 (86.7%)	182 (60.3%)	0.001
Low education (n = 622)^a^	38 (6.1%)	12 (3.7%)	26 (8.7%)	0.010
High education (n = 622)	413 (66.4%)	245 (75.9%)	168 (56.2)	< 0.001
Nulliparous (n = 584)	241 (41.3%)	133 (44.3%)	108 (38.0%)	0.122
Living in deprived area (n = 627)^b^	111 (17.7%)	37 (11.4%)	74 (24.4%)	< 0.001
Smoking in pregnancy (n = 554)	71 (12.8%)	35 (12.2%)	36 (13.5%)	0.650
Previous professional help for psychological problems (n = 624)	72 (14.9%)	147 (45.4%)	74 (24.7%)	0.000
Psychiatric medication (n = 468)	221 (35.4%)	14 (5.5%)	2 (0.9%)	0.007
BMI in kg/m^2^ (SD) (n = 603)	24.0 (5.0)	23.4 (4.2)	24.7 (5.7)	0.001

ªDutch Standard Classification of Education: 2006—Edition 2016/’17, CBS, Statistics Netherlands

^b^In the Netherlands ZIP code areas are used nationwide to classify the Social Economic Status of neighborhoods (Devillé, 2012)

### Association of Migrant Background with Complications and Healthcare

We found no differences between native Dutch women and women with a migrant background in terms of pregnancy related complications or pregnancy related healthcare consumption, except for gestational diabetes (Table 2). Gestational diabetes was more often found in women with migrant background [OR = 3.09; 95% CI = (1.51, 6.32)]. This association remained significant when adjusting for employment, education and BMI [OR = 2.29; 95% CI = (1.06, 5.00)]. Even when we added multiple co-variates, for example age, past mental history and research site, our results did not change (data not shown).

**Table 2:** Pregnancy-related complications and healthcare consumption by women with migrant background or native Dutch

	Total N (%)	Dutch N (%)	Migrant background N (%)	Migrant background vs Dutch OR, 95% CI	p value test OR = 1
Preterm delivery^a^ (n = 582)	74 (12.7)	45 (14.6)	29 (10.6)	0.70 (0.42, 1.15)	0.156
SGA^b^ (n = 544)	54 (9.9)	22 (7.7)	32 (12.5)	1.71 (0.97, 3.03)	0.065
Preeclampsia^c^ (n = 487)	27 (5.5)	15 (5.8)	12 (5.2)	0.89 (0.41, 1.94)	0.766
Gestational diabetes^d^ (n = 533)	40 (7.5)	11 (4.0)	29 (11.3)	3.09 (1.51, 6.32)	0.002
All assisted delivery^e^ (n = 627)	225 (35.9)	125 (38.6)	100 (33.0)	0.78 (0.57, 1.11)	0.146
Caesarean section (n = 531)	209 (39.4)	111 (40.2)	98 (38.4)	0.93 (0.66, 1.32)	0.674
Hospitalised during childbirth (n = 532)	214 (40.2)	115 (41.7)	99 (38.7)	0.88 (0.62, 1.25)	0.482
Hospital admissions during pregnancy (n = 512)	105 (20.5)	60 (22.6)	45 (18.3)	0.77 (0.50, 1.19)	0.233
Composite of complications (n = 528)	339 (64.2)	173 (62.7)	166 (65.9)	1.15 (0.80, 1.64)	0.445
Composite of hospitalisation (n = 627)	272 (43.4)	146 (45.1)	126 (41.6)	0.87 (0.63, 1.19)	0.380

^a^Birth before 37 weeks of gestation

^b^Small for Gestational Age: weight < p10 or bending off intra uterine growth curve ≥ 20%

^c^Hypertension in pregnancy with blood pressure ≥ 140/90 mmHg and proteinuria ≥ 300 mg/24 h

^d^After adjusting for employment, education and BMI, OR = 2.29; 95% CI = (1.06, 5.00)

^e^All assisted delivery: composite of caesarean section, emergency caesarean section and operative vaginal delivery

### Association of Migrant Background with Depressive and Anxiety Symptoms

Women with a migrant background showed more depressive symptoms, but not more anxiety symptoms, than native Dutch women (respectively EPDS = 6.58 ± 4.79 vs. 5.41 ± 4.45; p = 0.002, Cohen’s d = 0.25 and HADS-A: 4.66 ± 3.63 vs. 4.25 ± 3.34; p = 0.146; Cohen’s d = 0.12) (Table 3). However, after adjustment for employment and education the difference in depressive symptoms was no longer significant (difference in mean EPDS 0.71 points; Cohen’s d = 0.15; p = 0.070).

**Table 3:** Depression (EPDS) and Anxiety (HADS) severity scores by women with migrant background or native Dutch women

		Dutch Mean (SD)	Migrant background Mean (SD)	Difference (95% CI)	p value	R^2b^	Cohen’s d (95% CI)
EPDS	Unadjusted	5.41 (4.45)	6.58 (4.79)	1.17 (0.44, 1.90)	0.002	0.016	0.25 (0.10, 0.41)
	Adjusted^a^			0.71 (− 0.06, 1.47)	0.070	0.043	0.15 (− 0.01, 0.32)
HADS	Unadjusted	4.25 (3.34)	4.66 (3.63)	0.41 (− 0.14, 0.96)	0.146	0.003	0.12 (− 0.04, 0.28)
	Adjusted^a^			0.26 (− 0.33, 0.84)	0.389	0.010	0.07 (− 0.09, 0.24)

^a^Adjusted for being employed (yes/no), education (low, medium, high)

^b^Coefficient of determination in univariate regression model for unadjusted difference or multivariate model for adjusted difference

### Association of Depressive and Anxiety Symptoms with Complications and Healthcare

No association was found between depression and anxiety scores with complications during pregnancy in both native Dutch women and women with a migrant background, nor with complications separately, nor with the composite of complications [OR = 1.01; 95% CI = (0.98, 1.06) and OR = 1.00; 95% CI = (0.95, 1.06), respectively].

**Fig. 1:**
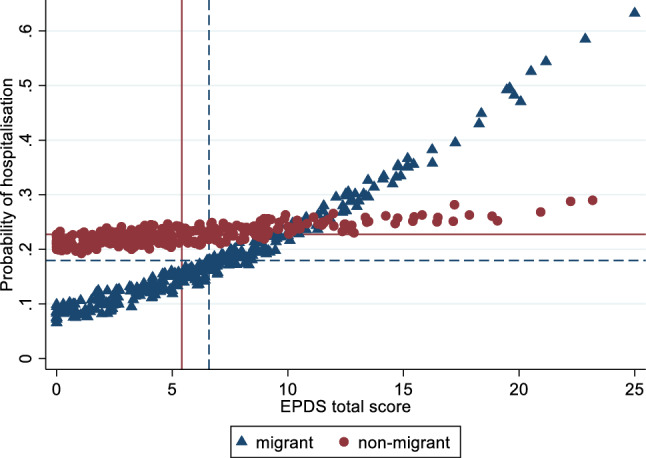
Graph showing the relationship between EPDS total scores and predicted probability of hospitalisation for both the migrant background and the non-migrant background group. Points are jittered to expose the distribution of observations across the EPDS total score axis. Vertical lines display mean EPDS total scores across groups and horizontal lines display mean probabilities of hospitalisation across groups, where dashed lines correspond to the migrant background group and solid lines correspond to the non-migrant background group

However, depression and anxiety scores were both associated with hospitalisation during pregnancy [OR = 1.06; 95% CI = (1.02, 1.11) and OR = 1.08; 95% CI = (1.02, 1.14)] per scale unit, respectively). This finding was not accounted for by differences in clinical or sociodemographic background factors. Women with a migrant background had a lower percentage of hospital admissions during pregnancy than native Dutch women (18.3% and 22.6%, respectively). However, when they reported more depressive symptoms the admission rate increased in contrast with the Dutch women where the admission rate remained unchanged, indicating that the relation between depression with hospital admission is stronger for pregnant women with a migrant background than for Dutch pregnant women [OR = 1.11; 95% CI = (1.01, 1.21), p = 0.028] (Fig. 1). This relation was not found for anxiety symptoms [OR = 1.07; 95% CI = (0.95, 1.21), p = 0.243].

## Discussion and Conclusions

In our cohort, pregnant women with a migrant background in the Netherlands did not have more pregnancy complications, except for gestational diabetes, compared to native Dutch women. Furthermore, women with a migrant background expressed more depressive symptoms than native Dutch women and if they had more depressive symptoms the probability of being hospitalised in pregnancy was higher.

Interestingly, our findings contradict many previous studies, which showed a correlation between women with a migrant background and more perinatal complications (Almeida et al., 2013; Ravelli et al., 2011).

There are some explanations for this contradiction. First, in many countries women with a migrant background have limited access to healthcare (Heaman et al., 2013; Ratcliff et al., 2015). Secondly, even with equal access to healthcare they tend to delay prenatal care and/or pay lesser prenatal visits (Chote et al., 2011; Kentoffio et al., 2016) thereby risking late detection of obstetric problems. Finally, differences in defining complications may have accounted for the different results. In our study we included the following complications: preterm birth, Small for Gestational Age (SGA), preeclampsia, gestational diabetes, caesarean section and all assisted delivery (caesarean section and operative vaginal delivery), while in some other studies stillbirth and neonatal mortality were also included (Ravelli et al., 2011). However, in our study the incidence of perinatal neonatal mortality (n = 6) was too low to be included in our analyses.

Also we did not find a significant difference in healthcare utilisation between women with a migrant background and women with a native background, which can be either explained by different healthcare systems (“free” versus paid healthcare) and/or by using different measures. In our study healthcare utilisation was defined by hospital admissions starting during pregnancy, while in other studies use of healthcare was defined as the total number of prenatal care visits (Posthumus et al., 2017).

This study also examined the relation between depressive or anxiety symptoms with complications and healthcare utilisation. Previous studies showed inconclusive relations. In a review and meta-analysis Grigoriadis found a small association between maternal depression during pregnancy and preterm birth (Grigoriadis et al., 2013) whereas others found only a relation between anxiety and depression and lower birth length (Broekman et al., 2014) or lower birthweight (Uguz et al., 2013) or did not find a relation at all with neonatal outcomes (Andersson et al., 2004). Here we also did not find a relation with neonatal outcomes. However, we found a small but significant association between depressive- and anxiety-symptoms with hospitalisation during pregnancy. An similar association between depressive symptoms and a lengthened predelivery stay on the obstetric ward was also found in the study of Palladino (Palladino et al., 2011). Depressive women with a migration background had a higher risk of hospital admission compared to depressive native Dutch pregnant women, but a lower risk of hospitalisation when not being depressed. One of the possible explanations for a higher risk of hospitalisation among pregnant women with a migrant background may be differences in coping strategy with depressive symptoms, possibly by somatisation, which induces hospitalisation (Morawa et al., 2017).

The high prevalence of depressive symptoms amongst pregnant women with a migrant background is consistent with the findings of most Dutch studies of non-pregnant people with a migrant background (de Wit et al., 2008; Haverkamp et al., 2015). Socioeconomic factors have been associated with symptoms of depression and anxiety among women with a migrant background (Malmusi et al., 2017), although they did not fully account for increased levels of depression (de Wit et al., 2008; Missine & Bracke, 2012).

This association may be related to how socioeconomic variables are defined and measured or that migrant background and socioeconomic factors are entangled, for example people with a migrant background have generally lower educations, and as a consequence are less often employed(Miszkurka et al., 2010). From that perspective, adjusting for socioeconomic factors may lead to overadjustment.

Finally, the migrant population in the Netherlands is somewhat difficult to compare with that of other countries, because of differences in the definition of migration and immigration history. In contrast to the study of Haverkamp et al. (2015), we did not find significant differences between the first and the second generation migrants, possibly due to a different constitution of participants and to small sample sizes.

## Strengths and Limitations of the Study

This study adds new data as we have investigated the relation between psychological distress and pregnancy complications and healthcare use in a country were healthcare is provided to all people, regardless of insurance or migration background (Posthumus et al., 2017).

Also, migration backgrounds were carefully documented and pregnancy-related data were directly obtained from medical records, which makes them more detailed and accurate than registry-based data. We also were able to adjust for multiple confounders.

There are also some limitations. One limitation is the selection of the study population thereby limiting generalizability of our findings. We performed our inclusion in a hospital setting and mainly included women who were referred because of (a history of) pregnancy complications, which resulted in a higher complication rate in our study sample than in the general population. The women were included at different gestational ages. Anxiety and depression will fluctuate over the course of pregnancy, also in pregnancies without complications, and in our study the symptoms were probably also dependent on time of occurrence or subsiding of the complications.

Also, we only included women from Amsterdam. However, including two hospitals from different neighbourhoods ameliorated the generalizability within Amsterdam. Also, in Amsterdam we could include more women with a migrant background which created a better possibility to find a differential effect. Moreover, our questionnaires were only provided in the English or Dutch language. Because some women were not able to read and write in Dutch or English they could not be included in our study. Yet, the number of women excluded for this reason was small (n = 59, 7.3%).

Although we had a reasonably large group of participants, the number was not sufficient to show differences between the various migrant groups. However, other studies have shown that the risk factors that different migrant groups have in common are more important in relation to pregnancy complications and healthcare consumption then the differences between them (Ravelli et al., 2011).

Another limitation is that we were not able to compare the excluded cases from our study sample as we did not have informed consent from the excluded women to access their medical record.

Finally, in some migrant backgrounds speaking about mental problems is not customary which could lead to underestimation of these problems among migrant women. However, the number of participating women with a migrant background (48.3%) was almost equal to the number of native Dutch participants (51.7%), indicating that reticence was probably not higher among women with a migrant background than in native Dutch participants.

## Conclusion

In this study of pregnant women visiting prenatal clinics of two large hospitals in Amsterdam, the Netherlands, we found that pregnant women with a migrant background did not experience more complications than native Dutch women, except gestational diabetes, and had an equal consumption of healthcare. Besides that, pregnant women with a migrant background experienced significant more depressive symptoms than native Dutch women, but not more anxiety symptoms. However, the difference in depressive symptoms might be (partially) explained by socio-economic factors like education and employment. Both depression and anxiety were associated with hospitalisation during pregnancy and women with a migrant background who suffered from depressive symptoms had a higher risk to get admitted in comparison to their native Dutch counterparts.

Future research should focus on additional factors contributing to depressive symptoms in migrant pregnant women, such as acculturation, social support and discrimination, and understanding the reasons for more frequent hospitalisations such as differences in coping. Addressing these factors may improve personalised care for pregnant women with a migrant background.

## Data Availability

Available from the corresponding author.

## Abbreviations

EPDS: Edinburgh Postnatal Depression Scale
HADS-A: The Hospital Depression and Anxiety Scale
BMI: Body Mass Index
CBS: Statistics Netherlands
